# Cervical squamous carcinoma cells are resistant to the combined action of tumor necrosis factor-α and histamine whereas normal keratinocytes undergo cytolysis

**DOI:** 10.1186/1471-2407-8-46

**Published:** 2008-02-07

**Authors:** Nicolae-Costin Diaconu, Jaana Rummukainen, Mikko Mättö, Anita Naukkarinen, Rauno J Harvima, Jukka Pelkonen, Ilkka T Harvima

**Affiliations:** 1 Department of Dermatology, University of Kuopio, Kuopio, Finland; 2 Department of Pathology, Kuopio University Hospital, Kuopio, Finland; 3 Department of Clinical Microbiology, University of Kuopio, Kuopio, Finland

## Abstract

**Background:**

Previous reports showed that mast cells can typically be found in the peritumoral stroma of cervix carcinomas as well as in many other cancers. Both histamine and TNF-α are potent preformed mast cell mediators and they can act simultaneously after release from mast cells. Thus, the effect of TNF-α and histamine on cervical carcinoma cell lines was studied.

**Methods and results:**

TNF-α alone induced slight growth inhibition and cell cycle arrest at G0/G1 phase in SiHa cells, but increased their migration. Histamine alone had no effect on cells. In addition, TNF-α and histamine in combination showed no additional effect over that by TNF-α alone, although SiHa cells were even pretreated with a protein synthesis inhibitor. Furthermore, TNF-α-sensitive ME-180 carcinoma cells were also resistant to the combination effect of TNF-α and histamine. In comparison, TNF-α or histamine alone induced growth inhibition in a non-cytolytic manner in normal keratinocytes, an effect that was further enhanced to cell cytolysis when both mediators acted in combination. Keratinocytes displayed strong TNF receptor (TNFR) I and II immunoreactivity, whereas SiHa and ME-180 cells did not. Furthermore, cervix carcinoma specimens revealed TNF-α immunoreactivity in peritumoral cells and carcinoma cells. However, the immunoreactivity of both TNFRs was less intense in carcinoma cells than that in epithelial cells in cervical specimens with non-specific inflammatory changes.

**Conclusion:**

SiHa and ME-180 cells are resistant to the cytolytic effect of TNF-α and histamine whereas normal keratinocytes undergo cytolysis, possibly due to the smaller amount of TNFRs in SiHa and ME-180 cells. In the cervix carcinoma, the malignant cells may resist this endogenous cytolytic action and TNF-α could even enhance carcinoma cell migration.

## Background

The most common form of cervical cancers of the uterus is squamous cell carcinoma (SCC), but the incidence of adenocarcinomas has been rising during the past few decades [1]. Even though virtually all SCCs and the overwhelming majority of adenocarcinomas are positive for human papilloma viruses (HPV) and HPV is the main causal factor for the development of cervical cancer [2]. The essential processes, that play a key role in the development of cervical squamous cell carcinoma, include regulation of cell apoptosis and proliferation, angiogenesis and immune surveillance. Inhibition of apoptosis, loss of cell cycle control, and stimulation of proliferation of an HPV-infected cell are essential features in the carcinoma growth [2].

Tumor necrosis factor-α (TNF-α) plays an important role during the inflammatory response and appears to be a key cytokine involved in antiviral, antibacterial and antiparasitic host defense mechanisms. The antitumoral effects of TNF-α have been shown to be the result of three different biological mechanisms: primarily hemorrhagic necrosis by TNF-α action on tumor endothelium, TNF-α immunomodulatory activity on immune effector cells, and a direct TNF-α mediated cytotoxic effect on tumor cells. However, paradoxically to its name and anticipated biological behaviour TNF-α can also have different effects which may promote tumor growth and metastasis [3,4]. The mechanisms for possible promotion of cervix carcinoma growth by TNF-α may include the stimulation of HPV-infected carcinoma cell growth in culture medium depleted from growth factors [5], induction of HPV-16 E6/E7 expression [6], and/or stimulation of epithelial tumor cell motility [7]. In addition, cervix carcinoma cells can be resistant to the cytolytic action of TNF-α or TNF-β and an additional stressor, such as treatment with a protein synthesis inhibitor or radiation, is needed for TNF-induced cytolysis [8-10].

Cancer cells are surrounded by stroma that contains numerous different cell types including the cells of the immune system. Therefore, numerous inflammatory mediators are acting simultaneously on cancer cells. Previously, an increase in cell lysis has been observed after the treatment of gynecologic tumor cell lines with interferon-γ and TNF-α [11,12]. A similar antiproliferative synergism has also been observed when treating gynecologic tumor cell lines with delta 12-prostaglandin J2 and TNF [13]. Also histamine has been reported to function synergistically with TNF-α when inducing intercellular adhesion molecule-1 expression in normal keratinocytes [14]. In addition, heparin can augment the growth inhibition of keratinocytes induced by TNF-α [15]. Histamine has been found to function synergistically also with interleukin-2 and this combination of mediators is under clinical trials in the treatment of different malignant diseases [16-18]. Furthermore, a cross-talk between histamine and interleukin-6 has been suggested in the regulation of melanoma cell growth [19]. Histamine, as well as TNF-α, has been found to function synergistically with chemotherapeutic drugs in the treatment of tumors and they appear to primarily target the tumor-associated vasculature [20]. Histamine and heparin are the essential and potent mediators residing in mast cell secretory granules, cells which are also a predominant source for TNF-α in epithelial cancer [21]. Also, mast cells have been found to be abundantly present in invasive carcinomas of the uterine cervix [22]. However, in addition to mast cells also cancer cells themselves can produce both histamine [23,24] and TNF-α [25-27].

Previous studies suggest that TNF-α may be able to act synergistically with histamine in the regulation of growth of carcinoma cells and keratinocytes, but functional evidence to support this hypothesis is very sparse. Both histamine and TNF-_ have previously been tested in clinical trials to treat cancer patients. Therefore, the well-established and HPV-16 positive SiHa carcinoma cell line derived from SCC of the uterine cervix was treated with TNF-α and/or histamine and subsequently the growth, viability, cell cycle, migration and invasion of SiHa cells were measured. Another carcinoma cell line, ME-180, was also used. In addition, the effect of TNF-α and histamine on the growth of normal keratinocytes was studied for comparison. Further, we measured and compared the expression of TNF receptors I and II in these cells and cervix carcinoma specimens. The results indicate that the combined action of TNF-α and histamine has profound cytolytic effects on normal keratinocytes but SiHa and ME-180 cells are resistant to these mediators suggesting that these malignant cells can escape from the control mechanism of TNF-α and histamine.

## Methods

### Chemicals

Minimum essential medium (MEM), McCoy's 5a medium, Keratinocyte-SFM medium, fetal calf serum (FCS), sodium pyruvate, sodium bicarbonate, non-essential amino acids and supplements for Keratinocyte-SFM medium were purchased from GIBCO™ (Life Technologies Ltd, Paisley, UK). Bovine serum albumin (BSA), 3-(4,5-dimetylthiazol-2-yl)-2,5-diphenyl tetrazolium bromide (MTT), emetine and diaminobensidine (DAB) were purchased from Sigma-Aldrich (St. Louis, MO, USA). Recombinant human TNF-α, goat anti-human TNFRI/TNFRSF1A polyclonal antibody, and goat anti-human TNFRII/TNFRSF1B (TNF-BPII) polyclonal antibody were from R&D Systems Europe Ltd (Oxon, UK) and rabbit polyclonal antibody against human TNF-α from HyCult biotechnology b.v. (Uden, The Netherlands). Hoechst 33258 (bis-benzimide) and propidium iodide are products of Molecular Probes Europe BV (Leiden, The Netherlands). Histamine diphosphate monohydrate was purchased from Fluka BioChemica (Buchs, Germany), Dulbecco's phosphate-buffered saline (PBS) and penicillin-streptomycin solution from Cambrex (Bio Science Verviers, Belgium), Vectastain ABC-Elite Goat IgG kit Vector PK-6103 from Vector Laboratories, INC. (Burlingame, CA, U.S.A.), NiCl_2 _and dimethylsulfoxide (DMSO) from Merck-Schuchardt (Munich, Germany). BD BioCoat™ Matrigel™ Invasion Chambers and control inserts were purchased from BD Bioscience (BD Bioscience Discovery Labware, Bedford, USA).

### Cultivation of normal keratinocytes and cervical squamous carcinoma cell lines SiHa and ME-180

Proliferating pure keratinocytes from human foreskin specimens were cultured under standard conditions (humidified atmosphere at 5% CO_2_, and 37°C) in Keratinocyte-SFM serum-free medium supplemented with epidermal growth factor (EGF), bovine pituitary extract (BPE), 100 U/ml penicillin and 100 μg/ml streptomycin [15,28]. The cells were passaged every 3–4 days and cells from passages 3^rd ^to 8^th ^with a viability of over 90% were used as measured by trypan blue exclusion method.

The SiHa carcinoma cell line derived from a 55-year-old japanese patient with grade II squamous cell carcinoma and the ME-180 cell line derived from a 66-year-old caucasian patient with a highly invasive squamous cell carcinoma in the uterine cervix were obtained from the American Type Culture Collection (Rockville, MD., USA). SiHa cells were cultured in minimum essential medium (MEM) supplemented with 10% fetal calf serum (FCS), 0.1 mM non-essential amino acids, 1.0 mM sodium pyruvate, 1.5 g/L sodium bicarbonate, 100 U/ml penicillin and 100 μg/ml streptomycin at 5% CO_2 _and 37°C. ME-180 cells were cultured in McCoy's 5a medium supplemented with 10% FCS. The SiHa cell line is reported to contain an integrated HPV-16 in the genome (1 to 2 copies per cell). ME-180 cells contain HPV DNA with greater homology to HPV-39 than HPV-18. The cells were passaged every 2–3 days. In every experiment, cells with a viability of over 90% were used.

### Fluorometric DNA Assay for Cell Growth

The cell growth by DNA content was determined using a fluorometric DNA assay as described previously [15,28]. Keratinocytes, ME-180 and SiHa cells were seeded in wells of a 24-well plate (Nunc, Roskilde, Denmark) with a density of about 15,000 cells/well (7,500 cells/cm^2^) using complete Keratinocyte-SFM medium for keratinocytes, complete McCoy's 5a medium for ME-180 cells, and complete MEM for SiHa cells. The optimal cell density was confirmed by using a variable number of cells. Next day, the medium was replaced with a fresh one (complete or incomplete Keratinocyte-SFM, McCoy's 5a medium or MEM). The adhered cells were treated with various concentrations of modulating agents or diluent control (PBS or PBS together with 1% bovine serum albumin) for 2–3 days and then a solution consisting of 0.04% sodium dodecyl sulphate and 8 M urea was added into the wells at 37°C for 1 h to dissolve the DNA of cells. Thereafter, 1.0 μg/ml of Hoechst 33258 was added and the fluorescence of the solution was measured using Spectra Fluor reader (Q-Lab Pty Ltd, Eagle Farm, Australia). The cultures and analyses were performed using quadruplicate wells and each experiment was performed at least four times.

### Determination of cytotoxicity and cell viability

Cell viability and cytotoxicity effects of different modulating agents were measured by the MTT assay as described [15,28]. Keratinocytes, ME-180 cells or SiHa cells were seeded in wells of a 96-microwell plate with a density of about 4,000 cells/well using complete Keratinocyte-SFM, McCoy's 5a medium or MEM, respectively. Next day, the medium was replaced with a fresh one (complete or incomplete Keratinocyte-SFM, McCoy's 5a medium or MEM). The adhered cells were treated with various concentrations of modulating agents for 6–8 h and then 0.33 mg/ml MTT in incomplete Keratinocyte-SFM was added into the wells at 37°C and 5% CO_2 _for 2 h. Thereafter, the MTT solution was removed and the formed intracellular dye was solubilized by incubating with DMSO for 15–20 min. The absorbance of the solution was measured at 550 nm using a micro-ELISA reader (SLT-Labinstruments GmbH, Salzburg, Austria). The cultures and analyses were performed using eight parallel wells and each experiment was performed at least four times.

### Determination of Cell Cycle

SiHa cells were seeded in 6-well plates using complete MEM medium. On the following day, the medium was changed to complete or incomplete MEM and histamine and/or TNF-α were added to the medium for 24 h. Thereafter, the conditioned medium containing possible detached cells was collected to a test tube. The attached SiHa cells were released by incubating in trypsin-EDTA for up to 10 min. After inactivating of trypsin with 10% FCS, the cell suspension was combined with spontaneously detached cells. The final SiHa cell suspension was centrifuged and washed with D-PBS. For fixation, the cells were suspended in 0.5 ml of cold D-PBS and the cells were added cautiously, drop by drop, to 5 ml of ice-cold 70% ethanol using continuous mix of the solution as described earlier [15]. After a minimum fixation for 1 day, the cells were centrifuged and suspended in D-PBS, treated with 0.15 mg/ml RNAse at 50°C for 1 h, and incubated in 16 μg/ml propidium iodide at 37°C for 2 h. Finally, the cells were analyzed using FACScan (BD Biosciences, USA) flow cytometer. The experiment was performed 3 times.

### Staining of TNF receptor I and II in keratinocytes and SiHa SCC cells

To study whether SiHa and ME-180 cells and normal keratinocytes are immunopositive for TNF receptor I and II, goat polyclonal anti-human TNFR I and TNFR II antibodies were used in immunocytochemistry [21]. First, keratinocytes, SiHa or ME-180 cells were seeded into the wells of a 4-well chamber slide (Nunc Lab-Tek™) in the presence of complete Keratinocyte-SFM, MEM or McCoy's 5a medium, respectively. After 2 or 3 days, the culture was stopped by removing the medium followed by washing the cells with PBS and fixing them in cold acetone for 10–15 min. Finally, the cells were stained immunocytochemically using Vectastain Elite ABC kit for visualizing the bound antibodies. The number of keratinocytes, SiHa or ME-180 cells positive for TNFRI and II were counted.

### Determination of keratinocyte growth and migration under high-calcium conditions

The growth and migration assay of normal human keratinocytes under high-calcium conditions has been described previously [15,28]. Briefly, metallic cylinders were placed (6 mm inner diameter) on the bottom of each well of an uncoated 6-well plate (Falcon, Becton Dickinson, Plymouth, UK). Before addition of cells, the wells and cylinders were equilibrated in 5 ml of complete Keratinocyte-SFM^® ^medium at 37°C and 5% CO_2_. Subsequently, about 30,000 keratinocytes, suspended in the same medium, were added cautiously into each cylinder. The cells were allowed to adhere onto the plastic surface overnight and practically complete confluence of keratinocytes was reached. After adherence of cells, the metallic cylinders were removed and Keratinocyte-SFM^® ^was changed to 5 ml of DMEM, 10% FCS, 100 U/ml penicillin and 100 μg/ml streptomycin, a medium which creates high-calcium conditions. Under these conditions, monolayer proliferating keratinocytes begin to differentiate and form a multilayer epithelium. After equilibration for 1–2 h, histamine and/or TNF-α were added in varying combinations and concentrations as described in Results. The medium and agents were changed every 2–3 days until the epithelium border almost reached the wall of the well. Thereafter, the medium was removed and 4% formaldehyde was added to the wells overnight for fixing the epithelium. Finally, the epithelium was stained with Mayer's hematoxylin overnight.

### Determination of SiHa cell migration and invasion by the in vitro transwell assay

BD Biocoat™ Matrigel™ Invasion chambers and control chambers (BD Biosciences Europe, Erembodegem, Belgium) for 24-well plates were used to study the effect of histamine and TNF-α on the migration and invasion of SiHa cells. For this, 2.5 × 10^4 ^SiHa cells were seeded into the transwells using incomplete DMEM. The lower well contained complete DMEM. After adherence of the cells at 37°C and 5% CO_2 _for 1 hour, histamine (0.1 or 1 mM) or TNF-α (10 or 50 ng/ml) alone or both in combination were added to the serum-free DMEM medium in the upper transwell chamber. The plates were kept at 37°C and 5% CO_2 _for 24 hours and thereafter the non-migrating and non-invading cells were removed from the upper surface of the membrane by "scrubbing". The cells on the lower surface of the membrane were stained with hematoxylin and counted under the microscope at 200× magnification using an ocular grid.

### Immunohistochemical staining method for TNF-α, TNFR I and II in uterine cervix specimens

Immunohistochemical staining for TNF-α and TNF receptors I and II [21] was performed on 4-μm-thick sections from formalin-fixed and paraffin-embedded tissue specimens from the uterine cervix collected and used anonymously from the archives of diagnostic specimens of Department of Clinical Pathology, Kuopio University Hospital. In order to stain TNF-α, the primary antibody was a rabbit polyclonal anti-human-TNF-α antibody (25 μg/ml). TNF receptors I and II were stained using goat polyclonal antibodies at the concentrations of 15 μg/ml and 10 μg/ml, respectively. The bound antibodies were visualized using the Vectastain Elite ABC kit (Vector Laboratories). As controls for cervix carcinoma specimens, sections from 10 uterine cervix specimens with nonspecific inflammatory changes were used. These diagnostic biopsies had been taken either during the routine Papanicolaou test or during surgery. These specimens were known to have non-specific abundant inflammatory lymphocyte infiltrates based on the histopathologic examination. None of the controls were found to have alterations due to HPV infection. The diagnostic carcinoma biopsies used in the study were from 8 patients with squamous cell carcinoma in the uterine cervix verified in the histopathologic examination.

Due to the large number of positive cells in cancer specimens, a scoring system was designed:

The scoring for TNF-α: "++" denotes numerous positive cell-groups showing strong staining; "+" denotes only moderate staining, mainly negative; "-" denotes that the cells are clearly negative.

The scoring for TNFR's: "++" – denotes numerous positive cell-groups (membrane staining for TNFRI and immunopositive cytoplasmic granules for TNFRII); "+" – denotes many weakly positive areas for TNFRI and few positive areas for TNFRII; "-" – denotes the cells are mostly negative but some weakly positive groups of cells are detected.

### Statistical Analysis

All data are expressed as the mean ± standard deviation (SD) or standard error (SE). For comparisons of the means, Student's t-test for paired samples was performed to test statistical significance (p < 0.05). Microsoft Excel software (Microsoft Company, Redmond, Washington, USA) was used in all analyses.

## Results

### TNF-α had a weak inhibitory effect but histamine had no effect on the DNA content of SiHa cells

**Figure 1:**
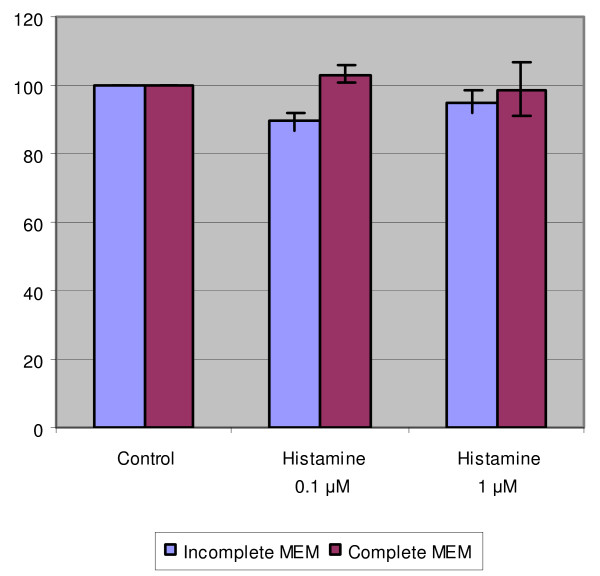
The effect of histamine on the DNA content of SiHa cells. The cells were grown in incomplete or complete MEM and treated with indicated concentrations of histamine. The data are expressed as the percentage of the fluorescence in the control wells. Each bar represents the mean of individual quadruplicate cultures ± SE (n = 4). "*" denotes p < 0.05 when compared with the control values.

Histamine alone (0.1 or 1.0 mM) had no marked effect on the DNA content of SiHa cells after cultivation in complete medium (Fig. 1). In incomplete medium, only slight but statistically non-significant inhibition was observed at both histamine concentrations. Nevertheless, the results did not reveal any stimulatory effect of histamine on SiHa cell growth.

**Figure 2:**
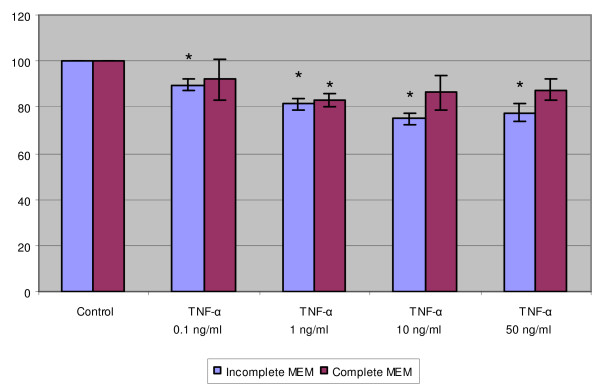
The effect of TNF-α on the DNA content of SiHa cells. The cells were grown in incomplete and complete MEM and treated with indicated concentrations of TNF-α. The data are expressed as the percentage of the fluorescence in the control wells. Each bar represents the mean of individual quadruplicate cultures ± SE (n = 4). "*" denotes p < 0.05 when compared with the control values.

TNF-α alone decreased slightly in a dose-dependent manner for up to 25% the DNA content of SiHa cells when they were cultured in incomplete medium (Fig. 2). Ten ng/ml TNF-α produced maximal inhibitory effect. In complete medium, the inhibition was not as apparent as in incomplete medium.

### The combination of TNF-α and histamine reduced in a cumulative manner the DNA content of normal keratinocytes but not that of SiHa and ME-180 cells

**Figure 3:**
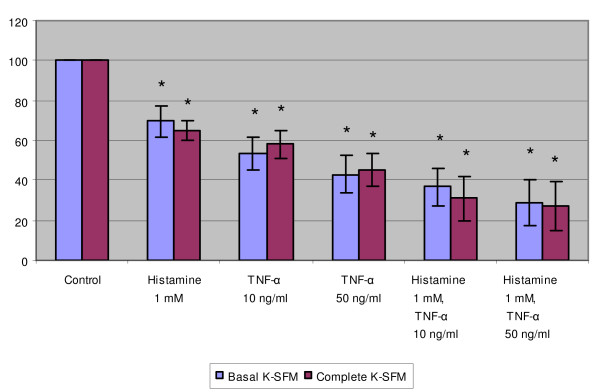
The effect of histamine (1 mM) and/or TNF-α (10 and 50 ng/ml) on the DNA content of normal keratinocytes. The cells were cultivated in incomplete or complete Keratinocyte-SFM medium and treated with indicated concentrations of histamine and/or TNF-α. The results are expressed as the percentage of the fluorescence in the control wells. Each bar represents the mean of four individual quadruplicate cultures ± SE. "*" denotes p < 0.05 when compared with the control values.

Histamine (0.1 and 1 mM) and/or TNF-α (10 and 50 ng/ml) were added alone or in combination to the cell cultures. Normal keratinocytes were compared to SiHa and ME-180 cells. Significant growth inhibition was found when keratinocytes were grown with histamine (1 mM) or TNF-α (10 or 50 ng/ml) (Fig. 3). Furthermore, when histamine and TNF-α were used in combination, the growth inhibitory effect on keratinocytes was found to be cumulative in both complete and incomplete Keratinocyte-SFM medium (Fig. 3).

**Figure 4:**
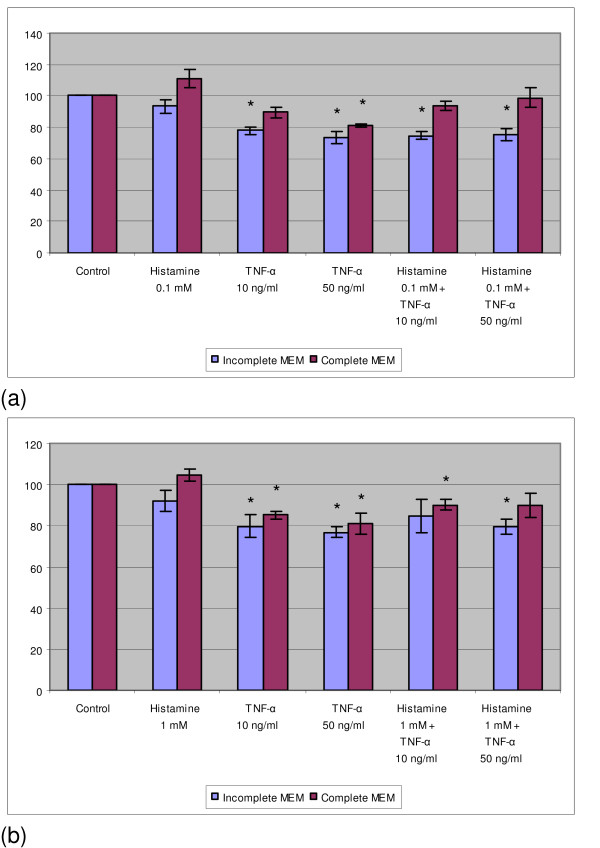
The effect of histamine and/or TNF-α on the DNA content of SiHa and ME-180 cells. SiHa cells were cultured with 0.1 mM histamine and/or TNF-α (a) or with 1 mM histamine and/or TNF-α (b) using incomplete or complete MEM. ME-180 cells were cultured with 1 mM histamine and/or TNF-α (c) using incomplete or complete McCoy's 5a medium. The results are expressed as the percentage of the fluorescence in the control wells. Each bar represents the mean of four individual quadruplicate cultures ± SE. "*" denotes p < 0.05 when compared with the control values.

In contrast to normal keratinocytes, histamine alone did not show any effect and TNF-α inhibited only slightly the growth of SiHa cells, especially in incomplete medium (Fig. 4a, b). Furthermore, of interest is the finding that the combination of histamine and TNF-α did not produce any additional inhibitory effect over that by TNF-α alone.

In order to clarify whether the ME-180 cell line known to be sensitive to TNF-α can respond further to the combination of TNF-α and histamine, these cells were cultured with these agents. As demonstrated in Fig. 4c, TNF-α alone inhibited significantly the growth of ME-180 cells but 1 mM histamine did not. However, the combination of TNF-α and histamine did not result in any further increase in growth inhibition.

### The combination of histamine and TNF-α was cytotoxic against keratinocytes but not against cervical squamous carcinoma cells

**Figure 5:**
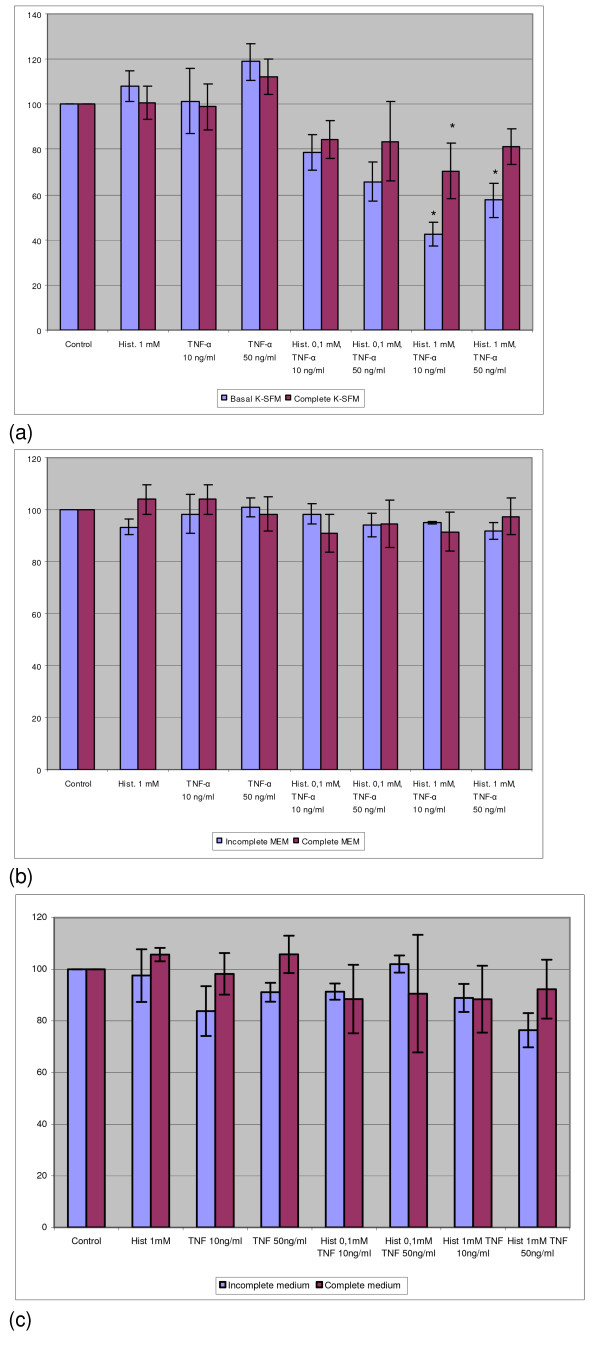
The cytotoxic effect of histamine and/or TNF-α on keratinocytes (a), SiHa cells (b) and ME-180 cells (c). The cells were cultivated in incomplete or complete medium and treated with indicated concentrations of histamine and/or TNF-α. The results are expressed as the percentage of the absorbance in the control wells. Each bar represents the mean of four individual cultures ± SE. "*" denotes p < 0.05 when compared with the control values.

The MTT assay was used to clarify whether histamine (0.1 and 1 mM) and/or TNF-α (10 and 50 ng/ml) had cytotoxic effects on SiHa, ME-180 cells and keratinocytes. Significant cytotoxicity was observed when keratinocytes were treated with the combination of 0.1 or 1 mM histamine and 10 or 50 ng/ml TNF-α in incomplete and complete medium (Fig. 5a). In addition, keratinocytes were treated first with either 0.5 mM histamine or 10 ng/ml TNF-α in complete medium for 1 day followed by replacing with a new medium and addition of increasing concentration of TNF-α (0, 1, 10 and 50 ng/ml) or histamine (0, 0.05, 0.5 and 1.0 mM), respectively, for another day. Consequently a similar cytotoxic effect in a dose-dependent manner could be seen in both culture systems, maximally at 10 ng/ml TNF-α or 0.5 mM histamine (not shown) indicating that a simultaneous presence of histamine and TNF-α is not required for cytotoxicity.

**Figure 6:**
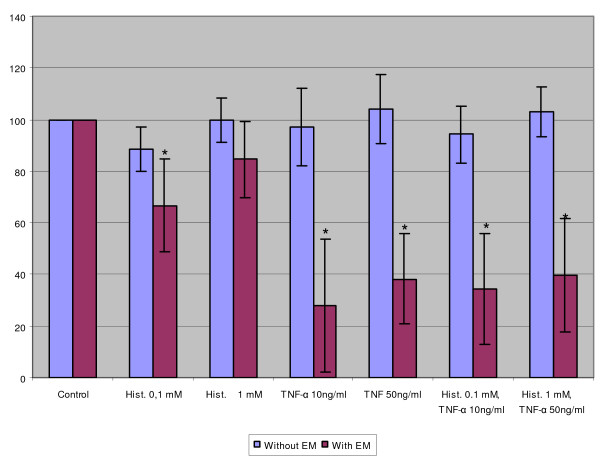
The effect of histamine and/or TNF-α on SiHa cells with and without inhibition of protein synthesis. The cells were pretreated with emetine and then cultivated and treated with indicated concentrations of histamine and/or TNF-α. The results are expressed as the percentage of the absorbance in the control wells. Each bar represents the mean of three individual cultures ± SD. "*" denotes p < 0.05 when compared with the control values.

In contrast to normal keratinocytes neither histamine nor TNF-α, or both in combination, could induce any marked cytotoxic effect on SiHa or ME-180 cells in complete and incomplete medium (Fig. 5b, c). Previously, the treatment of SiHa cells with the protein synthesis inhibitor emetine has been found to render the cells susceptible to the cytotoxic effects of TNF-α [8]. Therefore, emetine was also tested in this study to find out whether SiHa cells undergo cytolysis after treatment with emetine and subsequently with histamine and TNF-α. As demonstrated in Figure 6, the pretreatment of cells with 10 μM emetine for 18 hours resulted in an increase in the cytotoxicity by TNF-α alone. Also histamine appeared to be weakly cytotoxic. However, the combination of histamine and TNF-α did not show any increase in cytotoxicity when compared to TNF-α alone.

### The growth inhibition of keratinocyte epithelium is increased by the simultaneous action of TNF-α and histamine

**Figure 7:**
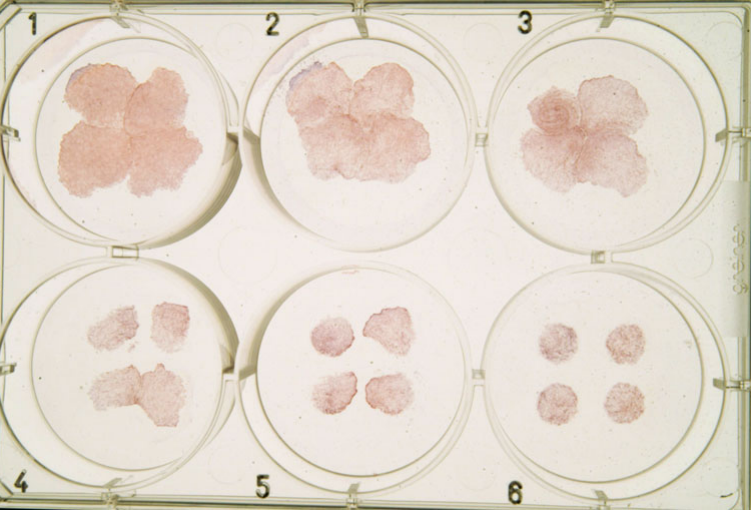
The effect of histamine and/or TNF-α on the growth of keratinocyte epithelium in a representative 3-day culture by using 10% fetal calf serum and DMEM as the culture medium. The following agents were added to the wells: well 1, diluent control; well 2, 0.1 mM histamine; well 3, 10 ng mL^-1 ^TNF-α; well 4, 50 ng mL^-1 ^TNF-α; well 5, 0.1 mM histamine + 10 ng mL^-1 ^TNF-α; well 6, 0.1 mM histamine + 50 ng mL^-1 ^TNF-α. Histamine (0.1 mM) or TNF-α (10 or 50 ng/ml) alone inhibits the growth of epithelium and the growth-inhibitory effect is increased when both agents are added to the same well.

The growth inhibition of keratinocytes induced by TNF-α and histamine was also confirmed under high-calcium conditions since carcinoma cells were also cultured in high-calcium medium. Histamine (0.1 and 1 mM) and TNF-α (10 and 50 ng/ml) were added into the wells of 6-well plates. As illustrated in Figure 7, both histamine and TNF-α inhibited the growth and migration of keratinocyte epithelium in high-calcium medium. Furthermore, the growth inhibition was increased when both agents were present simultaneously in the culture.

### TNF-α induced slight cell cycle arrest at G0/G1 in SiHa cells but the combination of histamine and TNF-α had no additional effect

In order to find out whether TNF-α (10 or 50 ng/ml), histamine (0.1 or 1 mM) or both in combination can modulate cell cycle or induce apoptotic cell death (subdiploid events in the cell cycle analysis), SiHa cells were cultured with these mediators for 1 day using both incomplete and complete medium. The adhered and detached cells were combined and analyzed by flow cytometry after propidium iodide staining. In three independent experiments with essentially similar results TNF-α at 10 and 50 ng/ml, histamine at 0.1 and 1 mM and the combination of those had no marked effect on the cell cycle in incomplete and complete medium, though TNF-α alone slightly induced cell cycle arrest at G0/G1 phase especially in incomplete medium. Neither histamine nor TNF-α or both in combination could induce apoptotic events above the control level (3.9% in incomplete and 0.9% in complete medium).

### TNF-α stimulated SiHa cell migration but the combination of histamine and TNF-α had no additional effect

**Figure 8:**
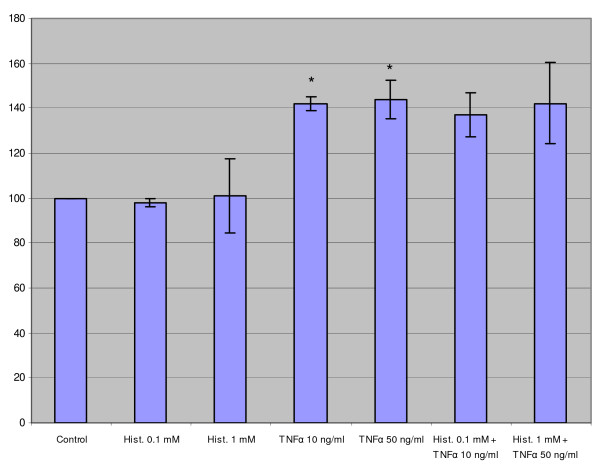
The effect of histamine and/or TNF-α on SiHa cell migration in the transwell assay. TNF-α significantly increased the cell migration but addition of histamine could not produce any additional effect over that by TNF-α alone. The results are expressed as the percentage of migrated cells compared to those in control wells. (n = 3; mean ± SD). The control is indicated as 100%. "*" denotes p < 0.05 when compared with control values.

In three independent assays, histamine and TNF-α alone or in combination were tested to find out whether they could influence the migration and invasion of SiHa cells in the transwell assay. When histamine alone (0.1 or 1 mM) was added to the transwell, no stimulatory or inhibitory effect on SiHa cell migration could be observed. Instead, a significant increase in the cell migration was found when the cells were treated with TNF-α. However, the combination of histamine (0.1 or 1 mM) and TNF-α (10 or 50 ng/ml) could not induce any additional effect over that by TNF-α alone (Fig. 8).

The effect of histamine and TNF-α alone or in combination on SiHa cell invasion was studied by using the Matrigel transwell assay. However, no significant changes took place when histamine, TNF-α or the combination of histamine and TNF-α where tested using the same mediator concentrations as in the migration assay, though the variation between experiments was marked. The ratio of the cell number of invaded cells to that of migrated cells in the control wells was 14.3 ± 4.9%.

### Normal keratinocytes were strongly immunopositive for TNFR I and II whereas SiHa and ME-180 cells were not

**Figure 9:**
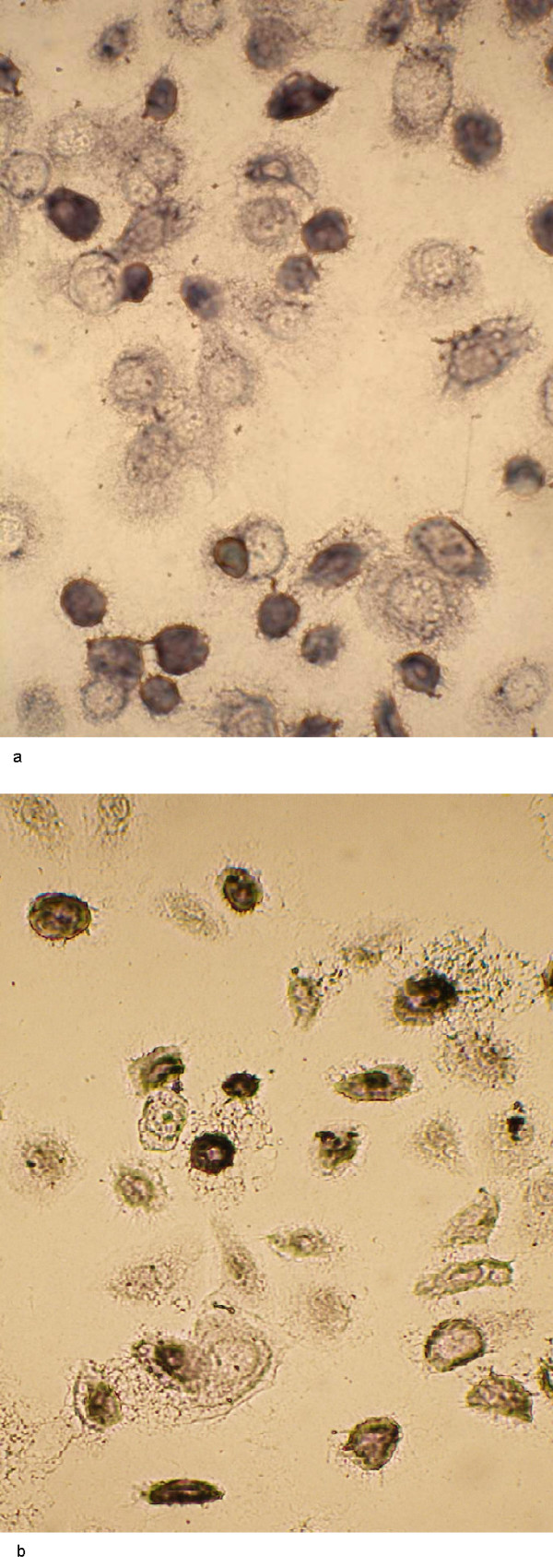
Immunocytochemical staining of TNFRI (a) and TNFR II (b) of normal keratinocytes.

**Figure 10:**
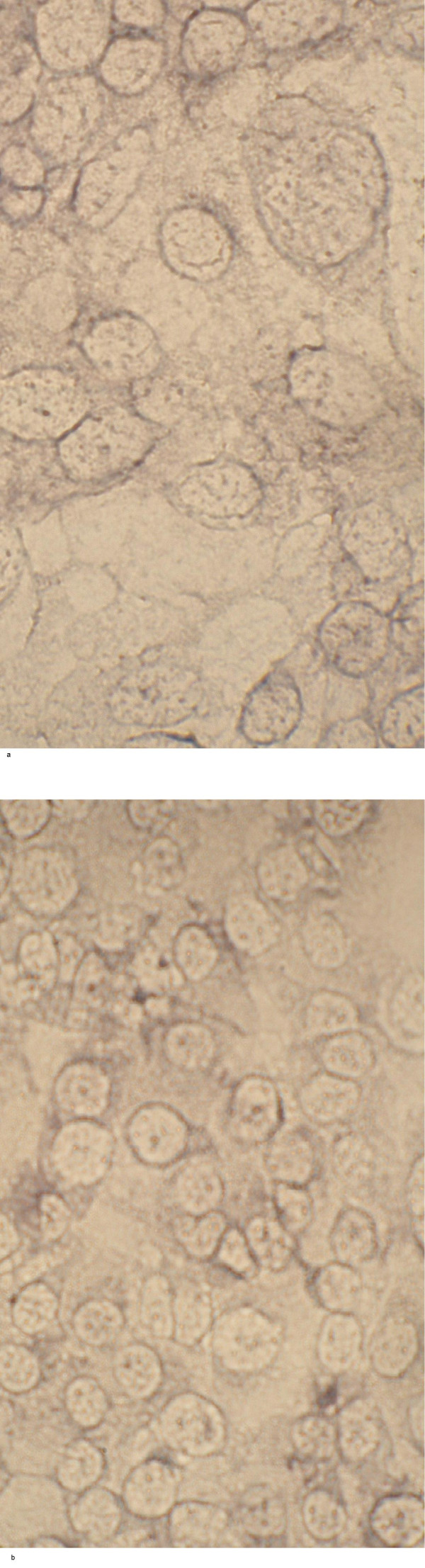
Immunocytochemical staining of TNFRI (a) and TNFR II (b) in SiHa SCC cells.

Chamber slides were used to cultivate SiHa and ME-180 cells and normal keratinocytes for the immunocytochemical staining of TNFRI and II. Three individual cultures were performed. In the case of keratinocyte culture, 52% and 36% of the keratinocytes showed immunoreactivity for TNFR I and TNFR II (Fig. 9), respectively. In contrast, SiHa cells displayed only weak immunopositivity and less then 1% of SiHa cells were found to be clearly immunopositive for TNFR I and II when over 1,000 cells were counted (Fig. 10). Similarly to SiHa cells, also ME-180 cells stained far less intensely to both TNFRs than keratinocytes (not shown).

### Numerous TNF-α positive cells in the cervix carcinoma

**Figure 11:**
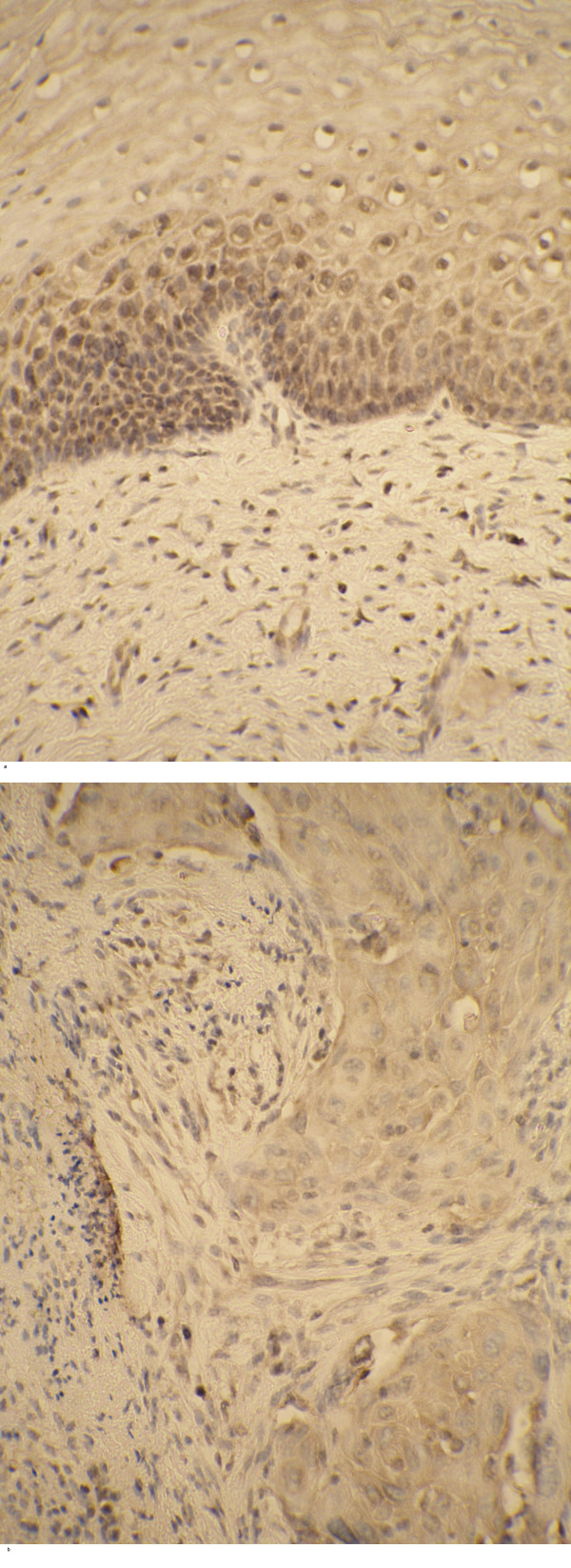
TNF-α positive cells in the cervix carcinoma – immunohistochemical staining of SCC specimens. Note: strong immunopositivity of the epithelial cells of control sample (a); TNF-α positive tumor cells and peritumoral stroma in cervix carcinoma (b).

TNF-α expression in 8 cervix carcinoma samples was semiquantitatively analyzed and compared to that in 10 control samples with nonspecific inflammatory changes. The results are expressed using a scoring system, both in the tumor and peritumoral stroma of carcinoma samples as well as in the upper subepithelial stroma and epithelium of control samples. Within the immunopositive cells, staining was predominantly localized to the cytoplasm in a granular fashion. Distribution of staining within the epithelium was remarkably similar and consistent in control samples: 7 specimens showed clear positivity and 3 samples very strong positivity in the basal and suprabasal epithelial cells (Fig. 11a). In cervix carcinoma samples, tumor cells and peritumoral stroma were found to be positive in 7 out of 8 cases. Strong immunopositivity was found in vascular endothelium and fibrous tissue (Fig. 11b).

### Carcinoma cells of the cervix seem to loose TNFI and II receptors when compared to epithelial cells of the control samples

The expression of TNFRI was evaluated in 8 samples of cervix carcinoma and in 11 samples from controls by immunohistochemistry. TNFRII was evaluated in 8 samples of cervix carcinoma and 10 controls (one section detached from slide). TNFRI- and TNFRII-positive cells were semiquantitatively analyzed (Table 1).

**Table 1:** The presence of TNF-α positive cells in the cervix carcinoma and control samples. Score of TNF-α

	Score of TNF-α			
	
		-	+	++
Carcinoma (no. of samples)	Tumor	1	6	1
	Peritumoral tissue	1	4	2
Control (no. of samples)	Epidermis	0	7	3
	Upperdermis	0	4	6

**Figure 12:**
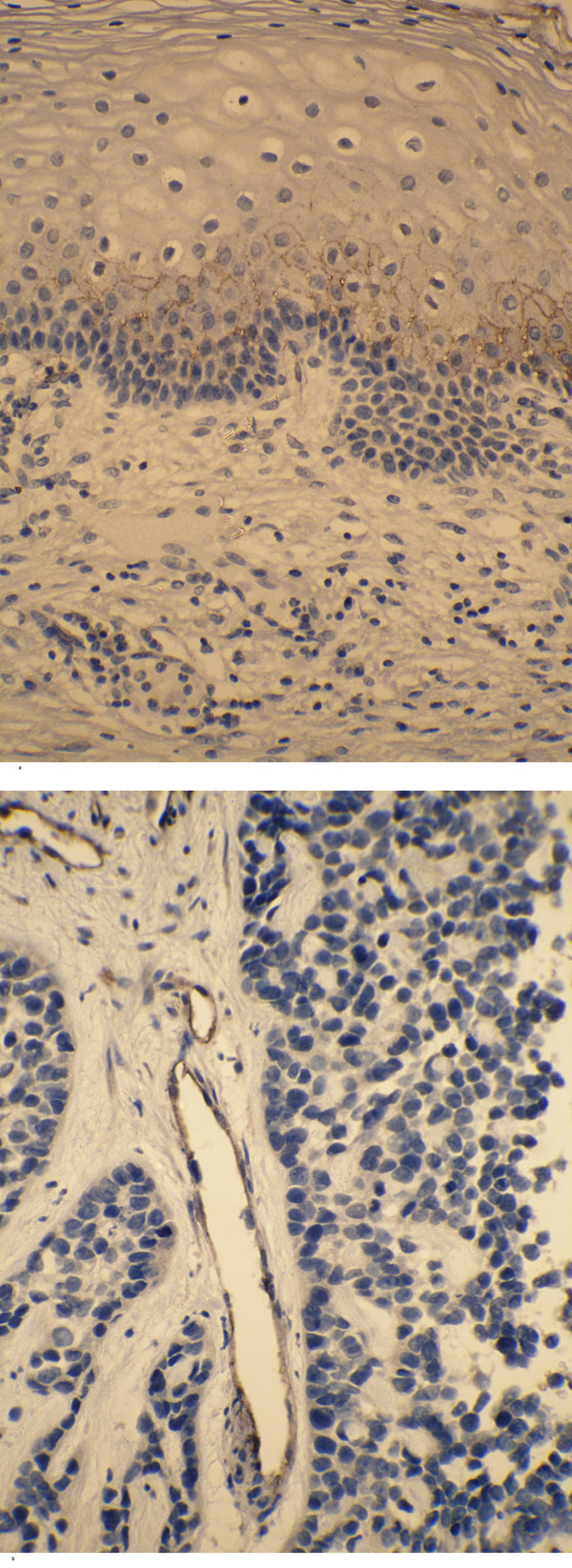
TNFRI positive cells in the cervix carcinoma. (a) – control sample; (b) – cervix carcinoma.

In the controls, 8 out of 11 samples displayed immunopositivity with TNFRI located in the basal layers of epidermis, whereas one sample showed positivity in the upper keratinocytes (Table 2). Half of the carcinoma samples had lost their positivity for TNFRI. TNFRII was shown positive in the upper layers of epidermis in all control samples, whereas tumor cells where found negative in two cases. Interestingly, both negative samples were also negative for TNFRI (Fig. 12).

**Table 2:** The presence of TNFRI- and TNFRII-positive cells in the cervix carcinoma and control samples

	Score of TNFRI			Score of TNFRII		
	
	++	+	-	++	+	-
Carcinoma (no. of samples)	1	3	4	1	5	2
Control (no. of samples)	1	8	2	7	4	0

## Discussion

Previously, histamine has been shown to inhibit the mitosis of keratinocytes by utilizing both H1- and H2-receptors [29]. When acting together, histamine and TNF-α have shown synergism in the induction of ICAM-1 on normal keratinocytes, an induction which probably requires H2-receptors [14]. On the other hand, histamine has been found to induce shedding of TNFRI from the plasma membrane and mobilization of the receptor from the Golgi apparatus in endothelial cells, and therefore histamine may transiently inhibit the action of TNF-α towards the cell [30]. In addition, histamine can suppress gene expression and synthesis of TNF-α via H2-receptors in peripheral blood mononuclear cells [31]. Thus, histamine together with TNF-α can induce a range of different effects on a cell.

In this study, the treatment of normal keratinocytes with histamine or TNF-α alone resulted in growth inhibition in a non-cytolytic manner. However, the simultaneous action of both histamine and TNF-α induced increased growth inhibition and cytolysis. Therefore, of interest is the previous finding on normal keratinocytes that the treatment of keratinocytes with histamine and TNF-α leads to increased ICAM-1 expression [14] and also to increased cytolysis. ICAM-1 expression in keratinocytes not only leads to T cell activation but also to increased lysis of keratinocytes by cytotoxic T cells [32]. Furthermore, the treatment of cultured keratinocytes with 1 μg/ml paraphenylenediamine for 3 hours has been found to induce ICAM-1 expression probably due to slight membrane damage, but 2 μg/ml paraphenylenediamine induced cytotoxicity [33]. Therefore, it is possible that during the simultaneous action of histamine and TNF-α, these mediators first activate and thereby induce ICAM-1 in keratinocytes but further activation causes cytolysis. To clarify this further, keratinocytes were first cultured with either histamine or TNF-α, i.e., in conditions which induce ICAM-1 but not cytotoxicity. After one day, the medium was changed and TNF-α or histamine, respectively, was added to the culture for one more day. As a result, increased cytolysis was detected by the MTT assay. Thus, preactivation of keratinocytes by histamine or TNF-α renders the cells more susceptible to the subsequent cytotoxic effects by TNF-α or histamine. Since this mechanism for keratinocyte death in low- and high-calcium conditions appears to be effective, it is possible that it is a previously unrecognized way for the tissue to control the growth of the epidermis or epithelium. Heparin binds efficiently TNF-α and augments the growth inhibition of keratinocytes induced by TNF-α [15]. Therefore, the combination of histamine, heparin and TNF-α may be even more potent for causing keratinocyte death.

TNF-α has controversial effects on cancer cells. It can cause cytolysis to some cancer cell lines and it has been used even in clinical trials in the treatment of cancer patients. On the other hand, it may stimulate cancer cell growth and therefore anti-TNF-α drugs have been proposed for cancer treatment [3,4]. Further complexity for the TNF-α effect on cells is caused by HPV viruses in cervical carcinomas. However, previous in vitro studies indicate that the TNF-α treatment can rather maintain the growth of cervical carcinoma cells, or even stimulate their growth in one study, than cause growth inhibition and cytolysis [5-8]. In order to induce cytolysis in these carcinoma cells by TNF-α, additional stressors are needed, such as protein synthesis inhibition or radiation [8,9]. In addition, the treatment with epidermal growth factor of ME180S cervical carcinoma cell line has been found to protect these TNF-α sensitive cells from TNF-α induced apoptosis [34]. In this study, up to 50 ng/ml TNF-α alone inhibited only slightly the growth of SiHa cells in a non-cytolytic manner especially in culture medium depleted from serum and growth factors. This inhibition can be attributed to the slight growth arrest at G0/G1 phase of the cell cycle found in the serum-free medium. Previously, TNF-α has been found to induce growth arrest at G0/G1 in normal keratinocytes and in HPV-16-immortalized keratinocytes [15,35]. Instead, no apparent changes in the cell cycle or mitotic regulatory proteins by TNF-α was observed in HPV-18-immortalized keratinocytes and in cervical carcinoma cells, including SiHa and HeLa cell lines [35]. However, TNF-α has been shown to induce slight inhibition in the proliferation of human SCC cell lines from hypopharynx, submaxillary gland, vulva and esophagus [7].

Previously, chemotactic migration of SiHa and CaSki cervical carcinoma cell lines to laminin-1 has been shown to be significantly decreased by TNF-α while migration towards type I collagen was increased [36]. In addition, TNF-α has a stimulatory effect on the migration of SW756 cervical carcinoma cells [37] and it induces motility of different epithelial tumor cell lines [7]. Thus, the present finding that TNF-α increased SiHa cell migration towards serum is in line with these previous findings. Nevertheless, TNF-α was not able to induce any apparent increase in the invasion of SiHa cells through Matrigel™, although the cells had some capacity to invade in the control wells. Previously, the invasion capacity of SiHa cells has been found to be relatively weak through Matrigel™ when compared with that of FaDu and A431 SCC cell lines [38]. The weak inhibition in the growth and the induction of slight cell cycle arrest at G0/G1 of SiHa cells by TNF-α were associated with increased TNF-α induced SiHa cell migration. A similar association between growth inhibition and increased motility has previously been observed in normal keratinocytes [39] and different epithelial tumor cell lines [7]. Therefore, even though carcinoma cell proliferation is slightly inhibited the increased motility of these cells induced by TNF-α may augment the spread of the tumor. Nevertheless, the intracellular molecular machinery recruited in these changes by TNF-α needs to be studied in the future. Furthermore, the spreading carcinoma cells may have some capability in escaping the immune surveillance since TNF-α has not been found to induce ICAM-1 and HLA class 1 and 2 antigens in SiHa and CaSki cells [40].

Histamine has been suggested to be involved in the regulation of cancer growth since mast cells can typically be found in increased numbers in the vicinity of tumors [21,22]. The concentration of histamine within mast cell secretory granules approximates 100 mM [41], and therefore histamine concentrations of up to 100–1,000 μM can be expected to occur in the microenvironment between mast cell and cancer cell. However, experimental evidence clarifying the role of histamine in cervix carcinoma is surprisingly sparse. Previously, histamine at up to 100 μM concentration has been found to stimulate the growth of HeLa and A431 SCC cell lines and chemotactic migration of HeLa cells through the H1 receptor [42]. Also, histamine at up to 100 μM concentration has been shown to inhibit the production of interferon-induced protein of 10 kDa in SCC15 squamous cell carcinoma line through the H2 receptor [43]. In this study, histamine at up to 1,000 μM concentration was found to inhibit the growth of monolayer proliferating keratinocytes as well as the growth of keratinocyte epithelium, a result which is in line with our previous findings [44]. In clear contrast to normal keratinocytes, histamine at 100 or 1000 μM concentration showed no effect on the growth, viability, cell cycle, migration and invasion of SiHa cells. Only weak cytotoxicity, if any, by histamine was noted when SiHa cells were pretreated first with emetine. Thus, histamine appears to have no marked regulatory role in SiHa cell growth and motility whereas it may control the growth of normal epithelium.

Even though normal keratinocytes, grown as monolayer or epithelium, underwent enhanced growth inhibition and cytolytic changes when exposed to TNF-α and histamine, no changes in SiHa cell growth, viability, cell cycle, migration and invasion by TNF-α and histamine were observed. The pretreatment of SiHa cells with emetine sensitized the cells to the cytolytic effect of TNF-α, as expected based on the previous publications [8,10,12]. However, even this additional stressor could not sensitise SiHa cells to increased cytolysis by the combined action of TNF-α and histamine. These results suggest that malignant SiHa cells have lost the capability to respond to this endogenous cytolytic controlling mechanism by TNF-α and histamine. The treatment of ME-180 cells with TNF-α alone resulted in marked growth inhibition in a non-cytolytic manner as expected. However, histamine alone had no effect. Similarly to SiHa cells, the growth and viability of ME-180 cells were not affected markedly by the combined action of TNF-α and histamine suggesting that cervix SCC cells are resistant to this cytolytic mechanism.

The effect of TNF-α is mediated to the cell via TNFRI and TNFRII receptors. The intracellular signal mechanisms are, however, complex since TNFR ligation can activate different signalling routes [4]. In this study, we chose to stain TNFRI and II immunocytochemically directly on chamber slides since proteolytic detachment of cells may cleave cell membrane structures and receptors. However, the disadvantage is that the method is rather semiquantitative and it may be difficult to clearly demonstrate weak TNFR staining. Nevertheless, both TNFRI and II immunoreactivities were markedly expressed in normal keratinocytes and therefore these cells can be supposed to respond to TNF-α in a normal fashion. Instead, untreated SiHa and ME-180 cells displayed only weak immunoreactivity of TNFRI and II and only some occasional cells appeared to clearly display these receptors. This clear difference may provide one explanation that normal keratinocytes respond to TNF-α and histamine in a cytolytic fashion whereas SiHa and ME-180 cells do not. However, additional investigation is needed to clarify this difference.

Previously, tumor cells of SCC from head and neck as well as from oral cavity have been shown to contain the immunoreactivity of TNF-α and TNFRI and II [25,26]. In addition, cervix carcinoma and SiHa cells have been shown to express TNF mRNA [45]. Since SiHa cells showed only weak TNFRI and II immunostaining these receptors as well as TNF-α were stained also in cervical carcinoma specimens. Cervix specimens showing non-specific inflammatory changes were chosen as controls since inflammation is a typical feature of cancers, too. However, these sample groups are not directly comparable. Nevertheless, only one cervix carcinoma specimen out of 8 revealed marked TNFRI or TNFRII immunopositivity. The same sample was also highly positive by TNF-α staining. In control samples, TNFRI showed slight positivity in 8 out of 11 cases, one case being strongly positive. For TNFRII, 7 cases were found strongly positive, whereas 4 were weakly positive. There were large areas among tumor cell sites showing no apparent staining of these receptors.

Thus, it appears that cervix carcinoma cells contain less receptors TNFRI and TNFRII when compared with the epithelium in samples with non-specific inflammation. Interestingly, a significant inverse correlation has been found between the expression intensity of 55-kDa TNFR and the velocity of tumor growth of oral SCC [25]. The peritumoral stroma contained TNF-α positive cells though the score for the subepithelial stroma of control specimens tended to be higher. However, both the carcinoma cells and control epithelial cells were immunostained in a similar intensity for TNF-α. Hence, TNF-α is present at least in some quantities in most of the carcinoma tissue specimens.

## Conclusion

In conclusion, the combination of TNF-α and histamine has a profound growth-inhibitory and cytolytic effect on normal keratinocytes. Instead, this combination showed no significant effect on the growth, viability, cell cycle, migration and invasion of SiHa cells suggesting that these cells have lost their capability to respond to this endogenous cytolytic mechanism, possibly owing to their lower TNFRI and TNFRII expression. Likewise, another SCC cell line, ME-180 was also resistant to the combined action of TNF-α and histamine. TNF-α alone induced slight growth inhibition and cell cycle arrest at G0/G1 phase in serum-free medium and increased the migration of SiHa cells. Since TNF-α was found to be present in the cervix carcinoma tissue it may have the capability to promote the spread of carcinoma cells. However, the expression level of TNFRI and TNFRII in the tumor cells of cervix carcinoma may not be sufficient for allowing cytolytic changes induced by TNF-α.

## Competing interests

The author(s) declare that they have no competing interests.

## Authors' contributions

NCD carried out the cell culture and immunohistochemical studies, participated in the preparation of the manuscript and performed the statistical analyses. JR and AN participated in the immunohistochemical analyses of cervical biopsies. MM and JP carried out the cell cycle assays. RH participated in the design and analyses of the study. IH conceived of the study and was the principal supervisor. All authors have read and approved the final manuscript.

## Pre-publication history

The pre-publication history for this paper can be accessed here:


